# Influence of Nanomaterials and Other Factors on Biohydrogen Production Rates in Microbial Electrolysis Cells—A Review

**DOI:** 10.3390/molecules27238594

**Published:** 2022-12-06

**Authors:** Nabil. K. Abd-Elrahman, Nuha Al-Harbi, Yas Al-Hadeethi, Adel Bandar Alruqi, Hiba Mohammed, Ahmad Umar, Sheikh Akbar

**Affiliations:** 1 Soil Fertility and Microbiology Department, Desert Research Center, El-Matareya, Cairo 11753, Egypt; 2 Department of Physics, Faculty of Applied Sciences, Umm AL-Qura University, Makkah 21955, Saudi Arabia; 3 Department of Physics, Faculty of Science, King Abdulaziz University, Jeddah 21589, Saudi Arabia; 4 Fondazione Novara Sviluppo, 28100 Novara, Italy; 5 Department of Chemistry, College of Science and Arts, Promising Centre for Sensors and Electronic Devices (PCSED), Najran University, Najran 11001, Saudi Arabia; 6 Department of Materials Science and Engineering, The Ohio State University, Columbus, OH 43210, USA

**Keywords:** Microbial Electrolysis Cells (MECs), bio-hydrogen production rates (Bio-HPR), nanomaterials, columbic efficiency (CE), cathode bio-hydrogen recovery (C Bio-HR)

## Abstract

Microbial Electrolysis Cells (MECs) are one of the bioreactors that have been used to produce bio-hydrogen by biological methods. The objective of this comprehensive review is to study the effects of MEC configuration (single-chamber and double-chamber), electrode materials (anode and cathode), substrates (sodium acetate, glucose, glycerol, domestic wastewater and industrial wastewater), pH, temperature, applied voltage and nanomaterials at maximum bio-hydrogen production rates (Bio-HPR). The obtained results were summarized based on the use of nanomaterials as electrodes, substrates, pH, temperature, applied voltage, Bio-HPR, columbic efficiency (CE) and cathode bio-hydrogen recovery (C Bio-HR). At the end of this review, future challenges for improving bio-hydrogen production in the MEC are also discussed.

## 1. Introduction

Hydrogen is considered one of the most promising and clean energy sources in the twenty-first century (future fuel) due to the fact that it contains many distinctive and effective properties, such as high energy conversion, large storage capacity, broad specific energy, renewable production and is environment friendly with zero emissions. It is widely used in industrial processes such as ammonia synthesis and petroleum refining, and thus hydrogen production is finding significant global interest [1,2].

Hydrogen is produced from fossil fuel sources with 96% of global hydrogen as follows: 48% from natural gas reforming, steam reforming or partial oxidation, 30% from naphtha reforming, and 18% from coal gasification [3]. However, hydrogen production in this way causes high-energy consumption and serious environmental pollution. Therefore, alternative ways to produce hydrogen from renewable environmental sources called biomass were searched for using biological methods [4].

Hydrogen production using biomass is considered better because of its high annual production and the reserves of its presence in the environment in large quantities, in addition to the ease of oxidation compared to hydrogen production using fossil fuel sources. The sources of biomass used in hydrogen production include agricultural waste, forest waste, domestic wastewater, industrial wastewater, carboxylic acids, polyols, sugars, wood waste, cellulose, lignin and microorganisms such as algae and bacteria [5,6].

There are several methods used to produce hydrogen fuel, called biological methods, including the photolysis method, photo fermentation method (PF), dark fermentation method (DF), double light fermentation method and microbial electrolysis method (MEC). Hydrogen produced from the use of biological methods is called bio-hydrogen [7].

Microbial electrolysis cell (MEC) is a biological method used to produce bio-hydrogen. Bio-hydrogen is produced in the MEC using multiple substrates, such as acetate, glycerol, glucose and various environmental wastes such as domestic wastewater and industrial wastewater. Bacteria and an applied voltage are used in MEC to decompose organic matter and produce electrons, protons and carbon dioxide. Bio-hydrogen in the MEC is produced by combining electrons with protons in a cathode chamber [8].

MEC was discovered and named for the first time as “electrochemical assisted hydrogen generation”, then “bio-catalyzed electrolysis”, “photoelectric generation”, and finally researchers agreed in their studies on the name “Microbial Electrolysis Cells’’ (MEC) [9]. MECs have many advantages that make them the best biological methods used for bio-hydrogen production. Firstly, environmental waste and renewable resources can be used as substrates for hydrogen production instead of fossil fuels. Secondly, bacteria can be used to transform various organic materials such as acetate, glucose, glycerol, cellulose, acetic acid, domestic wastewater and industrial wastewater to bio-hydrogen in the MEC. Thirdly, the productive efficiency of bio-hydrogen production using MEC is from 80–100%, compared to that of the electrolysis of water, which is about 65%, and that of dark fermentation, which is about 33%. In addition, MECs need an electric power supply of about 0.2–0.8 V, which is smaller than the voltage required for the electrolysis of conventional water (1.8–2 V) [10].

**Figure 1:**
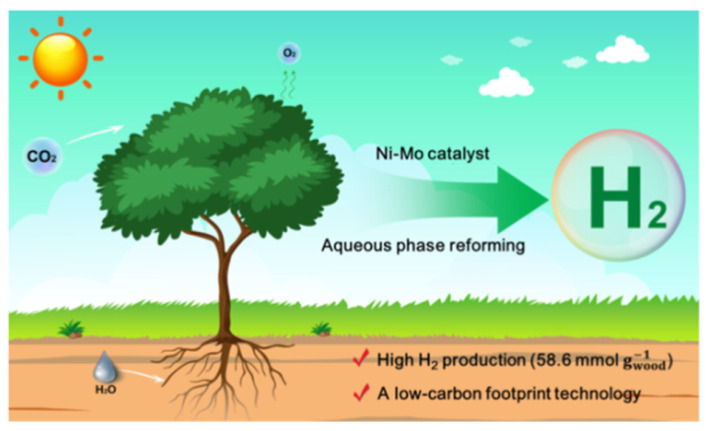
Low-temperature efficient hydrogen production from raw biomass on the Ni–Mo catalyst. reproduced with permission from ref. [12].

Nanomaterials have been used in many different engineering fields with the aim of finding a solution to technical problems and finding inexpensive solutions to improve and raise production and technical efficiency, due to the distinctive and different properties of nanomaterials that are not found in their bulk counterparts. The main objective of using nanomaterials in MEC is to improve the properties and performance of the basic components of MEC, which are the electrodes (anode and cathode), proton exchange membranes (PEM) and hydrogen production catalysts, or to replace the high-cost components with inexpensive components commensurate with the economics of hydrogen production in this way. Nanomaterials, especially when used to produce bio-hydrogen using MEC, are distinguished by their high electrical conductivity, high interaction surface area, high durability of materials, high catalytic ability to produce bio-hydrogen in MEC, biocompatibility with microorganisms and non-toxicity (Figure 1) [11,12].

This review focuses on previous studies illustrating bio-hydrogen production in MEC in three sections. The first section clarifies the idea of working and operating the MEC and its types (single chamber and double chamber) and the advantages and disadvantages of each. The second section discusses: the effect of using nanomaterials on the properties and performance of the anode, cathode and PEM, and thus on the performance of the MEC and bio-hydrogen production rates. The third section reviews the factors that affect the efficiency and operation of the MEC such as: substrate (sodium acetate, glycerol, domestic wastewater and industrial wastewater), pH, temperature and applied voltage.

## 2. Microbial Electrolysis Cells (MECs)

MECs are one of the bioreactors used to produce bio-hydrogen. Hydrogen fuel is a renewable energy source. Hydrogen is produced in the MEC using organic waste. Bacteria decompose and oxidize the organic matter in the substrates and produce electrons, protons and carbon dioxide. Electrons are released by bacteria in the anode chamber and then collect at the anode electrode. The electrons are then transferred from the anode electrode to the cathode electrode via external electrical circuits [13]. Protons are transferred from the anode chamber to the cathode chamber through the PEM in a double-chamber MEC and directly through solution in a single-chamber MEC. The protons combine with the electrons in the cathode chamber to produce hydrogen gas. However, the production of bio-hydrogen at the cathode is an endothermic reaction and therefore requires an applied voltage (0.2–0.8 V) [14]. The applied voltage (power supply) for bio-hydrogen production at the MEC (0.2–0.9 V) is much lower than for water electrolysis (1.23–1.8 V). The role of microbes is at play in producing electrons during the oxidation and decomposition of organic matter in substrates [15]. The basic reactions of bio-hydrogen production in MEC can be illustrated using acetate as a substrate for hydrogen production.

The following equations show the substrate decomposition reactions in the anode chamber and the hydrogen production reactions in the cathode chamber:

Anode: C_2_H_4_O_2_ + 2H_2_O → 2CO_2_ + 8e^−^ + 8H^+^

Cathode: 8H^+^ + 8e^−^ → 4H_2_

**Figure 2:**
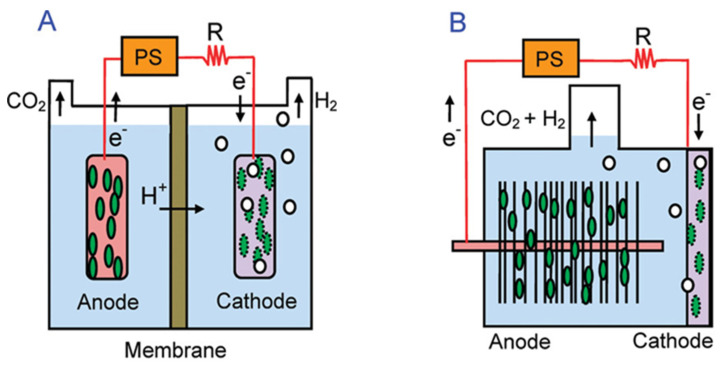
Schematics of (**A**) two–chamber (flat anode) and (**B**) single-chamber membraneless (brush anode) MECs. Reproduced with permission from ref. [16].

The rate of bio-hydrogen production in the MEC is influenced by several main factors that affect the efficiency of the performance of the MEC, which are the shape and type of the MEC (double-chamber or single-chamber), the type of materials from which the electrodes are made (anode and cathode) and the raw materials or substrate used to produce bio hydrogen (Figure 2) [16,17].

### 2.1. MEC Types and Shapes

Bio-hydrogen production rates are directly affected by MEC formation. MEC is divided into two main configurations: (1) double chamber MEC and (2) single chamber MEC. They have almost the same work principle. There is one difference between them, which is that there is only a PEM in the formation of the double-chambered MEC. There are many shapes of MEC, such as H-shaped, cubic, tubular or cassette-like [18].

#### 2.1.1. Double-Chamber MEC

**Figure 3:**
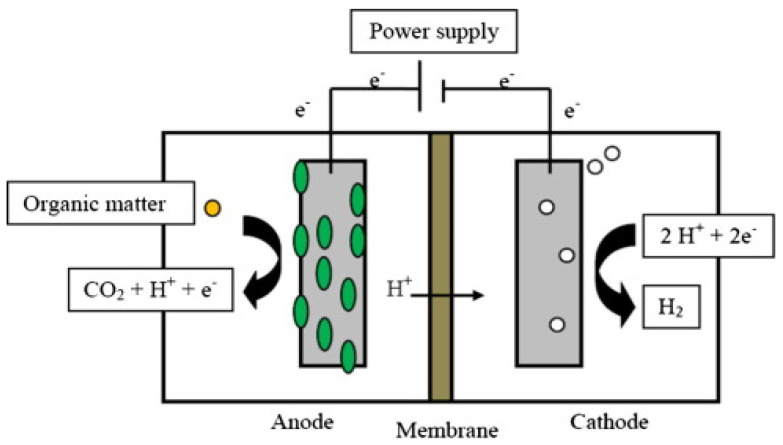
Schematic of double–chamber MEC operation and construction. Reproduced with permission from Ref. [19].

Double-chamber MEC is characterized by the presence of a PEM that separates the anode and cathode chambers. Double-chamber MEC takes an H-shape. The PEM is located in a channel connecting the anode and the cathode chambers. PEM is an important component of MEC because it prevents hydrogen diffusion from the cathode chamber to the anode chamber. Examples of membranes that have been used in MEC double chambers are proton exchange membranes (PEM), cation exchange membranes (CEM), and charge mosaic membranes (CMM). Bio-hydrogen is formed in the cathode chamber as a result of electron transfer from the anode to the cathode through the external circuit connecting the anode to the cathode, while H^+^ ions move across the membrane towards the cathode chamber (Figure 3) [17,19].

Bio-hydrogen is produced in MEC under anaerobic conditions for both the anode and the cathode chambers. The anode chamber contains bacteria that decompose the organic matter of the materials used to produce bio-hydrogen, and electrons are released. In the cathode chamber, the electrons combine with H^+^ ions forming hydrogen gas, which is collected in a tube above the cathode chamber [20].

The double-chamber MEC is characterized by its high efficiency in hydrogen production because it works on the migration of hydrogen ions from the anode chamber to the cathode chamber through the PEM and prevents the diffusion of oxygen hydrogen. A disadvantage of the double-chamber MEC is the higher manufacturing cost of expensive PEMs and the increased internal resistance [21].

#### 2.1.2. Single-Chamber MEC

The single-chamber MEC system was proposed and designed in 2008. Single-chamber MEC does not require an ion exchange membrane and both anode and cathode electrodes are located in one anaerobic chamber. Single-chamber MEC is not very different from double-chamber MEC in bio-hydrogen production reactions. In a single-chamber MEC, the organic matter in the substrate used to produce hydrogen is degraded by bacteria, releasing electrons, hydrogen ions and carbon dioxide. Electrons transfer from the anode to the cathode through the external circuit. The protons move directly to the cathode and combine with the electrons to produce bio-hydrogen [22].

The single-chamber MEC has the advantage of suitable design for practical applications due to its low manufacturing cost. The disadvantage of a single-chamber MEC is the easy diffusion of hydrogen to the anode, which leads to methane production and significant energy loss due to the discontinuation of anode reactions and methanogenic bacteria consuming the resulting hydrogen [23].

## 3. Application of Nanomaterials in MECs

The use of nanomaterials for bio-hydrogen production in MEC has been applied in many different ways, including thermal annealing, electrochemical anodizing, and electrodeposition. For example, in the thermal annealing method, the metal is exposed to a higher temperature than the normal temperature and then recrystallized. Fan et al. (2011) collected Au and Pd elements and exposed them to different temperatures ranging from 600 to 800 °C in order to convert them into nanomaterials [24]. In another study by Kim et al. (2018), the electrochemical oxidation method of Ti flakes with ethylene glycol electrolyte was used to obtain an external photo-anode of TiO_2_ array of nanotubes. The aim of this study was to modify the surface of the electrode to improve the reaction efficiency [25]. Several studies have shown that the electrodeposition method increases the Bio-HPR in the MEC [26].

Jayabalan et al. (2020, 2021) used a chemical deposition method to manufacture cathode catalysts from nickel molybdate (NiMoO_4_), nickel oxide (NiO) and cobalt oxide (Co_3_O_4_) nanoparticles [27,28]. Raney et al. (2021) also synthesized cathode catalysts from magnetite (Fe_3_O_4_) nanoparticles using a chemical deposition method [29].

The use of nanomaterials in MEC has been applied in different ways, including: (1) the addition of nanomaterials to the anode electrodes to improve the oxidation reactions of organic matter and electron transfer reactions; (2) using nanomaterials as cathode catalysts for the cathode electrode to improve bio-hydrogen production rates; and (3) the addition of nanomaterials to PEM to improve the membrane efficiency in bio-hydrogen production rates through the speed of proton exchange and resistance to biofouling of the membranes [30].

### 3.1. Anode Materials

The anode used in the manufacture of MEC should be characterized by a large surface area, good biocompatibility to help in the formation of the bacterial biofilm, good ability to resist corrosion, high electrical conductivity, good chemical and physical stability, non-toxicity, environmentally friendly status, availability and cheap cost [16].

Carbon materials are often used to make the anode electrode in bioreactor systems. Bio-electrochemical reactions depend on the activity of the anode. Since bacteria adhere to the surface of the anode and produce a biofilm, they supply energy to the anode and release electrons. The bacteria also break down organic matter into electrons, protons and carbon dioxide. Carbon-based materials are characterized by having a high surface area due to their high porosity, high electrical conductivity, availability in nature, and low cost, which makes carbon-based materials the most widely used anode materials in MEC [31]. Studies have proven that carbon materials used to manufacture the anode electrode help increase microbial colonization (biofilm) on the anode. The ability to form a biofilm improves the efficiency of releasing electrons. Carbon materials have been used to make anode electrodes in several ways, such as carbon fibers, carbon brushes, carbon felts, carbon meshes, carbon fibers and carbon foams. Most of the studies proved that the anode electrodes made of carbon fiber are highly effective in hydrogen production rates in MEC [32].

Graphite compounds are very similar to carbon compounds in that they are a good conductor of electricity, available and cheap. Therefore, graphite has become one of the most widely used materials for making anodes in MEC [33]. Several types of graphite electrodes have been manufactured, such as graphite granules, graphite rods, graphite brushes, graphite felts and graphite foams. Carbon compounds have a higher porosity than graphite compounds, which affects the percentage of bacterial adhesion that forms biofilms on the surface of the anode [34].

#### 3.1.1. Nanomaterials Used as Anodes

##### Metal/Metal Oxides Nanomaterials

The anode electrode is one of the main components of the MEC. The efficiency of microbial activity in the anode chamber, and thus the rates of bio-hydrogen production in the MEC, depends on the efficiency of the anode. Graphite is one of the materials used in the manufacture of the anode electrode. Fan et al. (2011) used the method of depositing nanomaterials on the anode electrode to improve the reaction efficiency, in which nanoparticles of Au and Pd with different shapes and sizes were applied to the graphite electrodes by the method of thermal annealing. Graphite electrodes decorated with nanoparticles were evaluated as anode electrodes in MECs. The results showed that graphite electrodes treated with Au nanoparticles gave up to 20 times more hydrogen production compared to normal graphite electrodes. This means that decorating the anode electrodes with nanoparticles can be one of the techniques used to improve the efficiency of the anode and thus increase Bio-HPR (Table 1) [24].

In a single-chamber MEC system, a photo-electrode is used. In a study to investigate the effect of nanoparticles on photo-electrodes, Kim et al. (2018) fabricated an array of TiO_2_ nanotubes and used them as catalysts for the Pt anode in single-chamber MEC [25]. During the operation of the MEC, the photo-anode made of TiO_2_ nanotubes was illuminated with a light source to simulate solar energy. The results showed a better performance of the photovoltaic rods, thus improving the MEC efficiency at Bio-HPR (1434 mmol/m^3^/h) compared to Bio-HPR under normal (dark) conditions (Table 1).

##### Carbon/Graphite Nanomaterials

Nanomaterials have been used to improve the efficiency of electrodes in bioreactor systems. Carbon nanotubes (CNTs) have been used to make the anode electrodes in MEC. CNTs have excellent electrical, mechanical, biological and thermal properties that make them ideal for making anodes [35].

**Table 1:** Nanomaterials employed for anode electrode and photo-anode electrode in MECs.

NanoMaterials/Size (nm)	D Structure	Method of NanoMaterials	Results	Ref.
Au (0.33 µm)	0	Thermal annealing	C D:74.4 µA/cm^2^	[24]
Pd (0.35 µm)	0	Thermal annealing	C D: 74.4 µA/cm^2^	[24]
TiO_2_ (Length:4.04–4.35 µm)	1	Anodization	C D: 0.371 mA/cm^2^ Bio-HPR: 1434 mmol/m^3^/h	[25]
CeO_2_–rGO	2	rGO (Modified Hummer’s and thermal reduction) CeO_2_–rGO (Polymerization and carbonization)	Bio-HPR: 5 m^3^/m^3^/d C Bio-HR: 95%	[36]

C D: current density, Bio-HPR: bio-hydrogen production rates, C Bio-HR: cathode bio-hydrogen recovery.

Pophali et al. (2020) fabricated a photo-catalysis-based anode using rGO nanosheets and CeO_2_ nanoparticles [36]. The rGO nano-sheets have a high electron-receptive capacity. This leads to the easy transfer of electrons and thus affects the efficiency of bio-hydrogen production. The same applies to the manufacture of nanocomposites for photo-catalysts CeO_2_ and rGO nanosheets (Table 1). The results showed that the photo-anode consisting of CeO_2_-rGO nanoparticles improved the working efficiency of MEC in bio-hydrogen production (cathode bio-hydrogen recovery (C Bio-HR) was 98%).

### 3.2. Cathode Materials

Various materials were used to manufacture the cathode in MEC, including carbon materials, stainless steel, platinum, nickel and titanium. The use of carbon materials as cathodes is disadvantaged by their low catalytic capacity for hydrogen evolution reactions (HER) due to their high potential [37]. Platinum is considered one of the best materials that has been used as a cathode due to its high catalytic ability. Results showed that hydrogen production rates were 14.54 ± 0.12 mL/L/day when using platinum as catalysts for the cathode at an applied voltage of 0.8 V. The use of platinum as a cathode is very rare and expensive. Platinum may also have a negative effect on the operating efficiency of MEC if it is contaminated with sulfides and cyanides [38].

The transition metal compounds of the first row of the periodic table may be excellent substitutes for platinum (Pt) in the manufacture of cathodes in MEC because of their high catalytic capacity and stability of chemical and physical properties, as well as that they are abundant in nature [39]. Nickel and stainless steel are materials that have been widely used so far as cathodes in MEC due to their excellent catalytic ability, abundant availability in nature, stable chemical and physical properties and low cost [16,40].

Stainless steel is a typical material for the manufacture of cathodes in MEC because it contains nickel, and therefore it is characterized by high catalytic ability, corrosion and rust resistance, high surface area and low cost. It is also an excellent alternative to high-cost Pt electrodes. Ni is more corrosion resistant than stainless steel, which is important for the electrode because it must be long lasting to be commercially viable [41].

#### 3.2.1. Nanomaterials Used as Cathodes

##### Cathode Catalysts by Metal/Metal Oxides Nanomaterials

Pt nanoparticles are often used as cathode catalysts in MEC, but due to the high price of Pt as a cathode, the use of some other nanomaterials has been researched as an alternative to Pt in terms of cost and effective ability to act as cathode catalysts [42]. Some of the nanomaterials that have been used as a substitute for Pt are highly conductive transition metals such as Ni and Cu as cathodic catalysts. In a study by Hrapovic et al. (2010), Ni nanoparticles with a relative size of 30–50 nm were used by coating them on a carbon paper cathode electrode using the electro-deposition method. The results showed that Ni nanoparticles had better catalytic performance than Pt in producing bio-hydrogen. The electro-deposition method of Ni nanoparticles can significantly reduce the operating and construction cost of MECs. The electro-deposition method can also be used with large cathode electrodes. In another study, Choi et al. (2019) evaluated the catalytic ability of the cathode using Ni and Cu nanoparticles. The results confirmed the ability of these nanoparticles to improve the catalytic efficiency of the cathode and thus increase the rates of bio-hydrogen production in the MEC [43,44].

Ni and Cu nanoparticles (17–20 nm) were used on carbon materials. Nanoparticles were fixed onto the carbon cathode electrodes by a spray method. The results showed that the nanoparticles of Ni and Cu have a catalytic efficiency of the cathode on the carbon electrode, which is less than that of the cathode made of Pt. In the stability tests of the catalyst, the results showed that the stability efficiency of both Ni and Cu is lower than the stability efficiency of Pt for hydrogen production. Considered in terms of price, catalytic ability and stability, Ni nanoparticles are the best alternative to Pt for hydrogen production in MEC [30].

One of the applications of MEC is that it can produce methane (CH_4_). For this purpose, Ni nanoparticles were also used as cathode catalysts. The deposition method of Ni nanoparticles with an average size of 40 nm was used on the cathode electrode made of granular activated carbon (GAC). A pair of Ni electrodes was dipped into an aqueous solution containing GAC. Then, plasma technology was used by exposing the electrodes that were dipped in the solution to by high voltage dipole pulses. The results showed the efficiency of the Ni nanoparticles in improving the cathode catalysis and accelerating the electron transfer from GAC to microorganisms [45].

In another study, Wang et al. (2019) used Pd nanoparticles as cathode catalysts instead of Ni nanoparticles. A bio-electrochemical deposition method was used to deposit Pd nanoparticles on a carbon cloth as cathode electrode. The results showed a significant improvement in the work of MEC in bio-hydrogen production [46].

Various transition metal oxides converted into nanoparticles were also used as catalysts for the cathode in the MEC for bio-hydrogen production. Kim et al. (2019) carried out a study aimed at evaluating the catalytic ability of metal oxide nanoparticles in bio-hydrogen production reactions in MEC. Ni_2_P nanoparticles with an average size of 7 nm were coated on carbon black using the solution phase method with carbon black particles (Vulcan XC-72R). The results showed that Ni_2_P catalysts gave Bio-HPR with a similar amount (0.29 L-H_2_/L-d) for Pt and Ni catalysts [47].

In another study by Chaurasia et al. (2020), an electroplating method was used to prepare Ni-CO-P nano-catalysts on stainless steel 316 and copper rods, which were used as cathodes. The results showed that the Ni-CO-P catalyst has a catalytic ability for the cathode electrodes, which resulted in an increase in bio-hydrogen production compared to using copper electrodes and stainless steel 316, although stainless steel is resistant to corrosion [42].

Taher (2019) [48] used nickel nanoparticles to fabricate a photocathode. An electro-deposition or spin-coating method was used by depositing NiFe_2_O_4_ nanoparticles on a glassy tungsten trioxide (WO_3_)-fluorescent tin oxide (FTO) electrode to fabricate photocathodes in MECs. The use of a combined photocathode with 1.5 wt.% nickel ferrite (NiFe_2_O_4_) nanoparticles resulted in a higher bio-hydrogen yield rate under visible light irradiation due to the highest Brunauer Emmett-Teller (BET) surface area with the smallest crystal size of 17 nm. In 2021, the cathode of nano-crystalline nickel molybdate (NiMoO_4_) was synthesized using a sono-chemical precipitation method using nickel (II) nitrate hexahydrate (Ni (NO_3_)_2_ 6H_2_O) and sodium molybdate (VI) dihydrate (Na_2_MoO_4_·2H_2_O) as precursors of Ni and molybdenum (Mo), respectively. The catalysts catalyzed with NiMoO_4_ nanoparticles showed better performance of Bio-HPR (0.12 L H_2_/L/day) and total hydrogen efficiency (11.96%).

The transition metal oxide nanoparticles were used as cathode catalysts in the MEC. Nickel (II) oxides (NiO) were prepared using nickel (II) chloride (NiCl_2_). Cobalt tetroxide (Co_3_O_4_) prepared with cobalt (II) chloride (CoCl_2_) was also evaluated using a chemical precipitation method as a cathode catalyst [27]. The compound nitrogen oxide was precipitated on nickel foam. The results of MEC operation showed that the use of Ni foam electrodes deposited with NiO and Co_3_O_4_ gave higher hydrogen production compared to using the bare Ni foam electrodes in MECs [30].

In another study to evaluate metal oxide nanoparticles, Rani et al. (2021) [29] used Fe_3_O_4_ nanoparticles with a size range of 12–28 nm as cathodic catalysts for bio-hydrogen production in MECs. The used Fe_3_O_4_ nanoparticles were deposited onto a graphite electrode and another made of carbon cloth using the deposition method. The results of the MEC operation showed that the carbon cloth electrode modified using iron oxide nanoparticles gave high Bio-HPR compared to using the unmodified carbon cloth electrodes.

Metal oxide nanoparticles with a three-dimensional (3D) structure were used as cathode catalysts and biofouling resistance for the cathode electrodes [49]. In a study by Kokko (2017), the electro-deposition method of molybdenum oxide (MoSx) nanoparticles was used on the carbon cathode electrode and thermal treatment was used to fix the nano-composites on the electrode. The results of the operation of the MEC showed that the Bio-HPR increased by 90% (0.26–0.39 m^3^/m^3^/day) [50].

NiO nanoparticle–loaded Y zeolite (NiO/Y zeolite) was used as a cathode catalyst in MECs for bio-hydrogen production in a study by Wang et al. (2019) to evaluate its catalytic ability and compare it with cathode catalysts made from Pt. Sodium hydroxide, sodium aluminate and amorphous silica were used to manufacture Y zeolite using a hydrothermal technique. The capillary impregnation method was used to obtain NiO/Y zeolite compounds. The results showed that the use of NiO/Y cathode catalysts led to a significant increase in Bio-HPR by 0.83 m^3^/m^3^/day with zeolite during the MEC operation compared with cathode catalysts made of Pt [49].

Zhao et al. (2019) also used transition metal oxide nanoparticles as cathode catalysts, such as NiO/MoO_2_/MoO_3_/C, to evaluate their bio-hydrogen production capacity in MECs and compare their catalytic ability with that of Pt. These nanoparticles were synthesized using the electro-deposition method directly onto the cathode made of carbon paper. According to the obtained results, the oxide nano-catalysts of these metals showed higher Bio-HPR than Pt, in addition to their strong durability [51].

Fang et al. (2021) synthesized a cathode electrode from Co, Ni and Fe, highly electrically conductive metals. These metals are effective catalysts in that they are inexpensive and have good catalytic capabilities. The combination of these metals improves electron transport and electrical conductivity. They fabricated the cathode catalysts in the MEC from a three-dimensional structured CoNi/CoFe_2_O_4_ nano-composite on a nickel electrode via the electro-deposition method. The results showed that 3D nanomaterials acted as excellent cathode catalysts for bio-hydrogen production (1.25 m^3^/m^3^/day) in MECs. In addition, they showed good stability and large surface area in the working of the cathode [26].

##### Cathode Catalysts by Carbon Nanomaterials

Carbon materials have a large surface area and good electrical conductivity, so they are used as electrodes. Choi et al. (2019) used carbon nanoparticles to manufacture cathode electrodes for bio-hydrogen production in MEC. Carbon nanoparticles were selected as cathode catalysts. The results showed that the use of carbon nanoparticles led to an increase in Bio-HPR by 47% [44].

The use of single-walled carbon nanotubes (SWCNTs) and multi-walled carbon nanotubes (MWCNTs) as cathode materials for MEC is characterized by their large surface area, good electrical conductivity and ease of operation. They can be used as cathodic catalysts to replace expensive Pt catalysts in bio-hydrogen-producing MECs [52]. However, the results did not show that using a cathode made of pure CNTs would produce similar or higher amounts of hydrogen than the cathode made of Pt. Therefore, to improve bio-hydrogen production using CNTs, the CNTs must be modified with other materials that have a high catalytic ability or are capable of creating synergistic effects with CNTs. Polyaniline can be used to modify MWCNTs due to its good tunable electrical conductivity, excellent stability and easy synthesis (Table 2) [53].

The catalytic potential of polyaniline carbon nanotubes is modified by adding MWCNTs to a hydrochloric acid (HCl) solution containing aniline monomers and dropping ammonium persulfate into the MWCNT dispersion. The results showed improved catalytic activity of polyaniline-modified CNT electrodes, increased electrochemical surface area; C Bio-HR 42% and Bio-HPR (0.67 m^3^/m^3^/d) were produced cost-effectively using MEC with polyaniline-CNT electrode [54].

Nitrogen-doped graphene (N-G), graphene oxide (GO) and reduced GO (rGO) are derivatives of graphene. The results of several studies showed that graphene derivatives can be used as cost-effective and high-performance cathodic catalysts for bio-hydrogen production in MEC (Table 2) [55,56].

Cai et al. (2016) used graphene to improve the catalytic ability of nickel foam cathode electrodes. The graphene-coated nickel foam cathode was prepared using a hydrothermal process. The results showed that the electrochemical and catalytic performance of the graphene-coated nickel-foam electrodes was improved and the Bio-HPR were significantly increased (1.31 L H_2_/L/d) compared to the cathode made of uncoated nickel-foam, and the performance is similar to that of the Pt catalyst electrode (Table 2) [57].

Dai et al. (2016) used a cathode electrode in his study that was made of carbon paper and coated with Mg(OH)_2_/GO nanoparticles. These nanoparticles were prepared using graphene oxide (GO) nano-sheets, MgSO_4_·7H_2_O as precursors and hydrazine hydrate as an additive. The results of the MEC operation showed that the carbon paper cathode electrode coated with nano-composites of Mg(OH)_2_/GO gave competitive results in bio-hydrogen production by 71% compared to the cathode electrode coated with Pt, in addition to strong stability during operation and its low cost [48].

In another study by Jayabalan et al. (2020), two types of graphene oxide nano-metal oxide rGO were prepared. NiO-rGO and Co_3_O_4_-rGO were used as cathodic catalysts for bio-hydrogen production in MEC using wastewater. Using a modified Hummer method, graphene oxide GO nanoparticles were synthesized. Using Ni and Co compounds, NiO-rGO and Co_3_O_4_-rGO nanoparticles were manufactured and then coated on nickel foam using the wet chemistry method. The results showed that the use of cathode catalysts for the nano-composites of Ni and Co with graphene oxides gave higher Bio-HPR compared to the uncoated nickel cathodes (Table 2) [55].

Other alternatives to Pt were also used as cathode catalysts for bio-hydrogen generation in MEC, including MoS_2_-based nanomaterials, which are suitable catalysts for hydrogen production because of their current exchange intensity and ability to absorb hydrogen. In a study by Rosenfeld et al. (2018) [58], MoS_2_ nanoparticles were used as cathode catalysts for hydrogen production in MEC. MoS_2_ nanoparticles were prepared by chemical methods from the conjugation of MoS_2_ particles (200 nm) with lithium. The results of the MEC operation for bio-hydrogen production showed that using MoS_2_ nanoparticles as cathode electrode catalysts gave a significant increase in Bio-HPR (0.133 m^3^/m^3^/day) compared to using Pt catalysts (Table 2).

**Table 2:** Nanomaterials employed for cathodic catalysts and cathodic photocatalyst in MECs.

Nanomaterials/Size (nm)	D Structure	Nanomaterials Preparation Method	Results	Ref.
Pt (Length: 7–20 µm)	0	Coating	C Bio-HR: 80.6%	[44]
Ni (Length: 7–20 µm)	0	Coating	C Bio-HR: 73.0%	[44]
Ni (Length: 30–50 µm)	0	Electro-deposition	C Bio-HR: 82%	[43]
Ni (Length: 40 µm)	0	Solution plasma	CH_4_ production enhancement: ~52.4%	[46]
Pt–Ni (Length: 7–20 µm)	0	Coating	C Bio-HR: 76.8%	[44]
Pt–Cu (Length: 7–20 µm)	0	Coating	C Bio-HR: 72.6%	[44]
Ni_2_P (7 µm)	0	Solution-phase	C Bio-HR: 65.5%	[47]
Ni–Co–P (Length: 33–35 µm)	0	Electro-deposition	C Bio-HR: 90.3%	[42]
NiFe_2_O_4_ (>17 µm)	0	Electro-deposition and spin coating	C D: 0.74 A/m^2^ Bio-HPR:288 mol/h/g	[48]
NiMoO_4_ (<50 µm)	0	Sono-chemical precipitation	C Bio-HR: 11.96%	[27]
NiO	0	Chemical precipitation	C Bio-HR: 27%	[28]
Pd (Length: 10–100 µm)	0	Bioelectochemical deposition	C Bio-HR: 65.5%	[46]
Co_3_O_4_	0	Chemical precipitation	C Bio-HR: 26%	[28]
Fe_3_O_4_ (Length: 12–28 µm)	0	Chemical precipitation	C D:15.2 mA/m^2^	[29]
Carbon (50 µm)	0	Coating	C Bio-HR: 47%	[44]
TiO_2_ nanorod (Length: 700 µm–Diameter: 40 µm)	1	Hydrothermal	Bio-HPR:4.4 µL/h	[59]
MoS_2_–TiO_2_ (Diameter: 100 µm)	1	Anodization and bioelectrochemical deposition	Bio-HPR:0.003 m^3^/m^3^/min	[60]
Mo_2_N	1	Hydrothermal synthesis and thermal annealing	C Bio-HR: 74%	[61]
CoP	1	Hydrothermal synthesis and thermal annealing	C Bio-HR: 34%	[62]
Polyaniline/MWCNT	1	Polyaniline deposition (Chemical oxidation polymerization)	C Bio-HR: 42%	[54]
		MWCNT: CVD (Chemical oxidation polymerization)	C Bio-HR: 56.7%	[53]
MoS_2_/CNT (Length: 1000 µm–Diameter: 73 µm)	1	CNT: CVD; MoS_2_ deposition: Hydrothermal	C Bio-HR: 49%	[63]
SWCNT	1	CVD	C Bio-HR: 38.9%	[44]
Polyaniline Nanofibers (Thickness: 50 µm)	1	Oxidizing aniline at a perchloric acid/dichloromethaneinterface	C Bio-HR: 79.2%	[52]
Polyimide Nanofibers (Thickness: 200 µm)	1	Electro-spinning	C Bio-HR: 32.4%	[64]
Graphene	2	Hummer’s and hydrothermal	Bio-HPR: 2.2 m^3^/m^3^/d	[57]
Mg(OH)_2_/graphene	2	GO nano-sheets: modified Hummer’s	C Bio-HR: 71%	[65]
		Mg(OH)_2_/graphene hydrothermal	C Bio-HR: 83% Bio-HPR: 0.63 m^3^/m^3^/d	[65]
NiO–rGO	2	Modified Hummer’s and chemical reduction	Bio-HPR: 4.38 mmol/L/d C Bio-HR: 21.2%	[55]
NiCo_2_O_4_–rGO		Modified Hummer’s and chemical reduction	Bio-HPR:3.66 mmol/L/d C Bio-HR: 18.2%	[66]
MoS_2_ (150–250 µm)	2	Chemical exfoliation by Li intercalation	Bio-HPR: 0.133 m^3^/m^3^/d C D: 0.6 mA/cm^2^	[58]
MoS_2_/N-doped graphene	2	MoS_2_ (Modified Hummer’s and chemical reduction)	Bio-HPR: 0.19 m^3^/m^3^/d	[67]
		MoS_2_–N–GO hydrothermal	Bio-HPR: 0.19 m^3^/m^3^/d	[67]
MoS_2_–GO	2	Solvothermal	Bio-HPR: 0.183 m^3^/m^3^/d	[56]
MoS_2_–Cu–rGO	2	GO (modified Hummer’s) MoS_2_–Cu–rGO hydrothermal	Bio-HPR:0.449 m^3^/m^3^/d	[68]
MoSx	3	Electro-deposition	C Bio-HR: 98%	[50]
Y Zeolites–NiO	3	Y zeolites: (hydrothermal process) Y Zeolite–NiO (incipient wetness impregnation)	Bio-HPR:0.83 m^3^/m^3^/d	[49]
NiO/MoO_2_/MoO_3_/C	3	Electro-deposition	C D: 37.5 A/m^2^	[26]
CoNi/CoFe_2_O_4_	3	CoFe_2_O_4_ (hydrothermal and calcination) CoNi/CoFe_2_O_4_ (unpolar and electro-deposition)	Bio-HPR:1.25 m^3^/m^3^/d	[26]
Activated carbon and Ni powder (0.5–1 µm)	3	Blending method	Bio-HPR: 0.28 m^3^/m^3^/d C Bio-HR: 98%	[69]

C D: current density, Bio-HPR: bio-hydrogen production rates, C Bio-HR: cathode bio-hydrogen recovery.

N-G nano-sheets possess many advantages that qualify them to be an alternative to cathode catalysts made of Pt, such as excellent electrical conductivity, good chemical stability and catalytic ability suitable for hydrogen production, and a large contact area suitable for charge transfer. MoS_2_ and GO nanoparticles were prepared using the chemical exfoliation method and modified Hummer’s method. Using a mixture of MoS_2_, GO and ammonia nano-sheets, 3D MoS_2_/N-G aerogels were synthesized via a hydrothermal process. The results showed that the MoS_2_/N-G air gel cathodes in the MEC gave distinct Bio-HPR of (0.19 m^3^/m^3^/day) with an applied voltage of 0.8 V, which was similar to the Bio-HPR obtained using a Pt catalytic electrode (Table 2) [56].

### 3.3. Nanomaterials Used as Membrane

The proton exchange membrane is an essential component of the double-chamber MEC. It is directly responsible for the production of bio-hydrogen in the cathode chamber. Many commercial membranes such as Nafion (proton exchange membrane), AMI-7001 (anion-exchange membranes) and NFR-PEM (nanofiber-reinforced composite proton exchange membrane) were used to produce bio-hydrogen in the MEC. The use of nanomaterials to improve the work and performance of MEC was not limited to stimulating the anode electrodes or stimulating the cathode electrode only. Several studies have been conducted to improve the performance of the PEM, also in order to increase Bio-HPR. One of the most important problems that affect the efficiency of the PEM is known as biofouling, which impedes the transfer of protons through the membranes from the anode chamber to the cathode chamber. Hydrogen is formed by combining a proton and an electron in a cathode chamber. Thus, the rate of hydrogen production depends on the rate of transfer of protons through the PEMs and the rate of speed of electron transfer [30].

Nanomaterials have also been used to fabricate PEM used in MEC. Park et al. (2017) fabricated a PEM from a sulfonated compound (arylene ether sulfone) (SPAES)/polyimide nanofiber (PIN). The thickness of the membrane fibers was about 200 nm using electro-spinning with a high voltage power source. Among the methods for manufacturing nano-proton exchange membranes that have been adopted are the methods of solution plasma, hydrothermal synthesis and chemical reduction [64].

Three-dimensional (3D) metal nanoparticles have been used to improve the efficiency of PEM as bio-fouling agents for the fabrication of membranes with high anti-fouling resistance. In a study by Park et al. (2021) using silver (Ag) nanoparticles to modify PEM to reduce the harmful effecting of biofouling on Bio-HPR, the PEM was coated with antibacterial Ag nanoparticles [70].

Coating the PEM with Ag nanoparticles had undesirable effects, including the release of Ag and the interference of proton transport that changed the chemical and physical properties of the membranes. Park et al. (2021) tried to reduce the harmful effects of coating Ag nanoparticles. Different coating methods were used, such as coating only Ag nanoparticles with ascorbic acid, along with coating with a polydopamine layer, followed by coating with Ag nanoparticles. Another method was used for membrane coating, which was an Ag nanoparticle coating, followed by coating with a polydopamine layer. The results of running the MEC using membranes that were coated with Ag nanoparticles and polydopamine in these two ways showed a high efficiency of bio-hydrogen production by 68.12% and reduction in biofouling to 80.74% compared to using the original membranes [30,70].

## 4. Factors Affecting Bio-HPR

The rate of bio-hydrogen production in the MEC is affected by several main factors that affect the efficiency of the performance of the MEC such as: substrate (sodium acetate, glycerol, domestic wastewater and industrial wastewater), pH, temperature, applied voltage and bacteria [17].

### 4.1. Substrates Used in the MEC

There are many organic substrates that have been used to produce bio-hydrogen in the MEC. Organic chemicals were used to produce bio-hydrogen, such as sodium acetate, glucose and glycerol. Environmental organic waste was also used, such as domestic wastewater and industrial wastewater. The types of these substrates directly affect the efficiency of the performance of the MEC, and thus the rates of bio-hydrogen production and the columbic efficiency (CE) [17,71]. Table 3 shows the effect of substrates on Bio-HPR in the MEC.

#### 4.1.1. Sodium Acetate

Sodium acetate (NaCH_3_COO) can be prepared in the laboratory or obtained as a by-product of dark fermentation. It is considered one of the most widely used basic substrates for the production of bio-hydrogen in the MEC in the laboratory, and is used as a good carbon source for bacteria. In a study by Rozendal et al., acetate was used as a substrate for bio-hydrogen production in a MEC with an anode chamber volume of 6.6 L at an applied voltage 0.55 V. The results showed that the bio-hydrogen production rates (Bio-HPR) reached 81 mL H_2_/L-d [72].

In another study by Jeon et al., to evaluate the catalytic ability of p-type polyaniline nanofibers (PANinfs) as a cathode material using acetate as a substrate at an applied voltage of 0.8 V they obtained Bio-HPR of 1.78 m^3^-H_2_/m^3^-d and CE of 98% [73].

In 2020, Hesibar et al. used acetate at a concentration of 6 g/L as a substrate for a single chamber MEC operation, with a working volume of 100 mL. The results showed a Bio-HPR of 0.31 ± 0.08 mmol-H_2_/L-d at an applied voltage of 0.8 V [74].

The type of MEC (single-chamber or double-chamber), the type of electrode material, as well as the applied voltage also affect bio-hydrogen production rates, in addition to the effect of the type of substrate used. Rozendal et al. evaluated the effect of electrodes and the use of acetate for bio-hydrogen production in a double-chamber MEC. Graphite and titanium were used as the cathode. The results showed a Bio-HPR of 0.02 m^3^-H_2_/m^3^-d. Acetate is the most widely used substrate so far in MEC, whether double or single-chamber, because it is a good carbon source for the growth and colonization of microbes on the anode electrode [38,74,75].

#### 4.1.2. Glycerol

Glycerol is a colorless, odorless and non-toxic chemical that has many medicinal uses and is indicated in many pharmaceutical industries as a good carbon source for the growth of different microbial species. Glycerol is obtained as a by-product during biodiesel purification technologies. Each 100 L of biofuel produces about 10 L of glycerol. Glycerol was used as a basic material for bio-hydrogen production in MEC. There are many previous studies that show the importance of glycerol as an organic compound used to produce bio-hydrogen [71].

Preliminary studies of using glycerol as a raw material for bio-hydrogen production in MEC indicated that hydrogen production rates were low. Another experimental study reported the use of glycerol added to domestic wastewater as a substrate for bio-hydrogen production in a double chamber of MEC with the use of Pt as a cathodic catalyst. The results showed a Bio-HPR of 0.77 mol/mol-glycerol at an applied voltage of 0.5 V [76].

When using crude glycerol as a substrate for bio-hydrogen production in a single chamber MEC, a graphite fiber with a Poly tetra fluoro-ethylene (PTFE) diffusion layer and 5 mg Pt/cm^2^ was used as a cathode at an applied voltage of 0.8 V. The results showed a Bio-HPR of 0.46 L-H_2_/L-d and CE of 55% [74].

#### 4.1.3. Domestic Wastewater

MEC is usually used in the production of bio-hydrogen and the treatment of domestic wastewater and industrial wastewater at the same time. In 2007, Ditzig et al. were able to use wastewater as a substrate in MEC for bio-hydrogen production using graphite granules as anodes. The results showed a Bio-HPR of 0.0125 mg-H_2_/mg at an applied voltage of 0.41 V and CE of 26% [17].

Several studies have been carried out to produce bio-hydrogen using wastewater as a substrate in MEC. The use of domestic wastewater as a substrate for hydrogen production is characterized by its high content of organic matter and the fact that it is a good source of carbon used by bacteria and an available, always renewable and inexpensive source. Converting domestic wastewater into an effective fuel such as hydrogen contributes to solving the problem of environmental pollution, global warming and climate change [77].

#### 4.1.4. Industrial Wastewater

Industrial wastewater has attracted great interest from researchers in terms of using it as a substrate in MEC for bio-hydrogen production, as it contains high organic matter, especially industrial wastewater from various food industries. Industrial wastewater was used for the manufacture of potatoes as a substrate in single-chamber MEC containing an anode electrode made of graphite fibers and cathode electrode made of Pt. The results showed that the Bio-HPR reached an average of 0.74 m^3^/m^3^/day at an applied voltage of 0.9 V [17].

In a study by Maron et al., six different types of industrial wastewater were used as a substrate in MEC for bio-hydrogen production. The industrial wastewater from fruit juice, cheese, sugar, paper and spirits industries was examined. Industrial wastewater was used, each individually, in six double MEC chambers, with anode chamber volume of 400 mL. All of these substrates were fermented before being used in the MEC to enhance the microbial activity of biofilm formation. The results showed that the Bio-HPR was 1609 mL/g-COD and the COD removal rate was 79% at an applied voltage of 0.2 V [78].

The eventual goal of using environmental wastes such as domestic wastewater and industrial wastewater as substrates in the MEC for bio-hydrogen production is to produce efficient and renewable fuels from harmful environmental pollutants, treat domestic wastewater and industrial wastewater at the same time and reduce the harmful environmental effects of the presence of these wastes in the environment without treating them [79].

**Table 3:** Effect of substrates on performance of MEC.

Substrate	MEC Types	Anode	Cathode	Results	Ref.
Sodium acetate	Double chamber	Graphite felt	Titanium mesh	CE: 53% Bio-HPR: 0.02 m^3^/m^3^/d	[74]
		Carbon paper	Pt + carbon Paper	CE: 92.0% Bio-HPR: 7.86 m^3^/m^3^/d	[80]
		Graphite felt	Nickel foam	CE: 90% Bio-HPR: 50 m^3^/m^3^/d	[81]
		Graphite brush	Carbon cloth	CE: 53% Bio-HPR: 0.08 m^3^/m^3^/d	[82]
		Carbon fiber	Stainless steel (304)	CE: 91% Bio-HPR: 0.53 m^3^/m^3^/d	[83]
	Single chamber	Graphite brush	Pt + carbon cloth	CE: 78% Bio-HPR: 3.12 m^3^/m^3^/d	[74]
		Graphite fibers	Stainless steel (304)	CE: 78% Bio-HPR: 1.7 m^3^/m^3^/d	[75]
		Carbon cloth	Pt + carbon cloth	CE: 53% Bio-HPR: 0.31 m^3^/m^3^/d	[74]
Glycerol	Double chamber	Carbon cloth	Platinum	CE: 53% Bio-HPR: 0.77 m^3^/m^3^/d	[71]
		Graphite brush	Stainless steel + carbon cloth	CE: 53% Bio-HPR: 0.05 m^3^/m^3^/d	[79]
		Graphite fiber	Pt + graphite fiber	CE: 35% Bio-HPR: 0.021 m^3^/m^3^/d	[22]
	Single chamber	Graphite brush	Pt + garbon cloth	CE: 99% Bio-HPR: 2.01 m^3^/m^3^/d	[75]
		Graphite brush	Pt + graphite fiber	CE: 55% Bio-HPR: 0.46 m^3^/m^3^/d	[84]
		Graphite fiber	Pt	CE: 55% Bio-HPR: 0.46 L/L/d	[74]
Domestic wastewater	Double chamber	Carbon paper	Carbon paper	CE: 26% C Bio-HR: 37.5%	[77]
		Graphite granules	Stainless steel	CE: 26% Bio-HPR: 0.0125 mg-H_2_/mg	[17]
	Single chamber	Graphite felt	Nickel	CE: 38% C Bio-HR: 45%	[77]
Sugar industry	Double chamber	Graphite	Nickel	CE: 45.1% Bio-HPR: 0.817 m^3^/m^3^/d	[85]
		Graphite	Stainless steel (304)	CE: 54.5% Bio-HPR: 1.329 m^3^/m^3^/d	[85]
		Graphite	Nickel foam	CE: 59.1% Bio-HPR: 1.594 m^3^/m^3^/d	[85]
		Graphite Plate	Nickel foam/NiO-rGO	CE: 54.67% Bio-HPR: 4.38 m^3^/m^3^/d	[55]
		Graphite Plate	Nickel foam/Co_3_O_4_-rGO	CE: 56.64% Bio-HPR: 3.66 m^3^/m^3^/d	[55]
		Graphite	NiMoO_4_/nickel foam	CE: 58.72% Bio-HPR: 4.28 m^3^/m^3^/d	[27]
	Single chamber	Graphite fiber	Pt	Bio-HPR: 0.74 m^3^/m^3^/d	[17]

C E: columbic efficiency, Bio-HPR: bio-hydrogen production rates, C Bio-HR: cathode bio-hydrogen recovery.

### 4.2. pH

MEC operating efficiency and Bio-HPR depend on the pH of the anode and cathode chambers. The pH directly affects the oxidation and reduction reactions in the anode and cathode chambers. Increasing the pH leads to an increase in the transfer or leakage of cations instead of protons across the PEM and thus the cathode electrode becomes alkaline while the anode becomes acidic, which causes energy loss due to a decrease in the speed of electron transfer, and thus low rates of bio-hydrogen production. Bacteria are very sensitive to rates of change in pH. The change in pH leads to a change in the growth of microbial activity and thus affects the oxidation of the substrate, the transfer rates of electrons and protons, and thus affects the rates of hydrogen production in the cathode chamber, since microbes grow efficiently at neutral pH 7. Previous studies reported that low pH in the cathode chamber improved Bio-HPR, while high pH in the anode chamber led to the accumulation of protons and prevented their transfer to the cathode chamber through the PEM. This caused a decrease in Bio-HPR [86].

Merrill et al. [87] found that lowering the pH in the cathode chamber improved the performance of the MEC by decreasing the solution resistance and cathode overvoltage. Table 4 shows the effect of pH on Bio-HPR in MEC.

### 4.3. Temperature

Temperature is one of the important factors that directly affect the performance of the MEC. Bacterial activity depends entirely on temperature. The optimum temperature for the growth of most microbes is from 35–40 °C. If the optimum temperature is provided for the growth of bacteria in the anode chamber, this improves the metabolism, increases the enzymatic activity of bacteria, formation of the biofilm on the anode electrode and generation of electrons and improves the electron transfer process and generation of energy, which increases the ability of bacteria to analyze the organic matter of the substrate. Omidi et al., in their study, showed that a 31 °C operating temperature is the most efficient In terms of Bio-HPR [71].

As a result, the operating temperature of the MEC must be maintained according to the optimum temperature for bacterial growth (30–40 °C) used in bio-hydrogen production to avoid consumption of the resulting hydrogen and the production of methane instead of hydrogen [90]. Table 5 shows the effect of temperature on Bio-HPR in MEC.

### 4.4. Applied Voltage

The minimum applied voltage used to operate the MEC is 0.2 V, in order to break the thermodynamic barrier to practically produce bio-hydrogen in the MEC. This leads to reducing the efficiency of the overall process in the cathode chamber and increasing Bio-HPR. Most of the previous studies demonstrated an increase in Bio-HPR by increasing the applied voltage to operate the MEC. The applied voltage used to produce bio-hydrogen in the MEC ranges between 0.2–0.8 V [91]. Table 6 shows the effect of applied voltage (V) on Bio-HPR in MEC.

### 4.5. Bacteria

There are many species of bacteria that are used in the production of bio-hydrogen in MEC, such as *Shewanella* sp., *Geobacter* sp., *Desulfuromondales* sp., *Pseudomonas* sp., *Clostridium* sp. *Geobacter sulfurreducens*, *Shewanella oneidensis*, *Geoalkalibacter* sp., *Dysgomonas* sp., *Bacteroides* sp., *Rhodopseudomonas palustris*, *Pseudomonas aeroginosa* and *Lactobacillus* sp. Bacterial species belong to the families Clotridiaceae, Comamonadaceae, Rhodocycaceae, Pseudomonadaceae, Geobacteraceae, Eubacteriaceae and Comamonadaceae [102].

The biological conversion of organic matter within the MEC into chemicals such as hydrogen occurs using the metabolic activity of some bacterial species. Bacteria that can produce electrons are called electroactive bacteria, and have an important role in transferring electrons from organic matter to the electrodes [103]. Table 7 shows the effect of some bacterial species on Bio-HPR in MEC.

## 5. Future Challenges to Improve Bio-Hydrogen Production

Hydrogen is considered the fuel of the future as one of the clean and renewable energy sources. Several methods for hydrogen production have been developed, including chemical and biological methods. Chemical methods have many disadvantages, such as high energy consumption and environmental pollution. Biological methods, such as photo fermentation, dark fermentation and MEC, are considered environmentally friendly methods. However, bio-hydrogen production faces some of the difficulties of any other renewable energy system, such as the high production cost versus production yield. MEC is one of the best biological methods used to produce bio-hydrogen. MEC is characterized by the production of pure hydrogen, does not cause any type of environmental pollution and contributes to the disposal of environmental pollutants by using domestic wastewater and industrial wastewater as a raw materials for bio-hydrogen production. Several studies have been conducted that show the importance of MEC in bio-hydrogen production and its role in wastewater treatment. There are many challenges that determine the efficiency of the operation of the MEC and hydrogen production rates, so the following aspects must be considered in future research to overcome and meet these challenges.

Theoretical studies and practical applications should be used to develop the efficiency of the operation of the MEC. The focus in these studies should be on the engineering of the construction and installation of the MEC, especially the basic components such as the anode, cathode and proton exchange membrane. The study of factors affecting the efficiency of MEC performance, such as electrode materials, substrate, pH, temperature and applied voltage, is necessary.

The materials used in the manufacture of the electrodes must be characterized by high conductivity, high surface area, good chemical and physical stability, an environmentally friendly status, good corrosion resistance and low cost to contribute to reducing production costs.

The anode materials must possess a good biocompatibility in the formation of bacterial bio-films and must be non-toxic to the bacteria used in the production of bio-hydrogen. Additionally, the materials used in the manufacture of the cathode must have a high catalytic ability.

Many previous studies proved the importance of using nanomaterials in developing the efficiency of MEC. Therefore, the optimum concentrations of nanoparticles that can be used to manufacture the electrodes must be studied. The use of nanomaterials had a good effect in increasing the electrical conductivity of the anode and the catalytic ability of the cathode.

PEM is an important and influential component of bio-hydrogen production in MEC. Therefore, nanomaterials must be used in the appropriate concentrations in the manufacture of PEM to increase the efficiency of proton exchange.

## 6. Conclusions

MEC is one of the best biological methods used to produce bio-hydrogen because of its high efficiency in converting substrates into hydrogen (80–100%) compared to water electrolysis (65%) and dark fermentation (33%). Using the double chamber MEC in bio-hydrogen production is better than using the single chamber MEC because of the purity of the hydrogen produced and the presence of a PEM that prevents the consumption of the resulting hydrogen and prevents bacteria from producing methane. Domestic wastewater and industrial wastewater are good substrates for bio-hydrogen production. The optimum temperature for bacterial growth and operation of the MEC is 31 °C, and the ideal pH = 7 and applied voltage (0.2–0.8 V). The use of carbon materials (carbon–graphite) as materials for the manufacture of the anode is better than metals because of their high porosity, which allows the formation of a biofilm and an increase in the rates of electron emission, and thus an increase the Bio-HPR. The use of nickel and stainless steel as cathode catalysts is a good alternative to Pt because of their high catalytic ability and low cost. The use of nanomaterials in the MEC improves the efficiency of the anode reactions and the catalytic ability of the cathode electrode and increases the transfer rates of protons through the PEM and thus improves the Bio-HPR.

## Figures and Tables

**Table 4:** Effect of pH on performance of MEC.

pH	Substrate	Applied Voltage (V)	Results	Ref.
4.5	Industrial wastewater	0.2	Bio-HPR: 0.41 m^3^/m^3^/d	[88]
5	Domestic wastewater	0.6	CE: 40% Bio-HPR: 0.52 m^3^/m^3^/d C Bio-HR: 32% COD removal: 32.5%	[87]
		0.8	CE: 60% Bio-HPR: 1.1 m^3^/m^3^/d C Bio-HR: 45% COD removal: 30.5%	[89]
5.5	Rich straw fermentation		Bio-HPR: 1.01 m^3^/m^3^/d	[88]
6	Industrial wastewater		Bio-HPR: 1.52 m^3^/m^3^/d	[88]
6.5	Rich straw fermentation		Bio-HPR: 2.46 m^3^/m^3^/d	[88]
7	Domestic wastewater	0.6	CE: 28% Bio-HPR: 0.13 m^3^/m^3^/d C Bio-HR: 2.5% COD removal: 30.2%	[86]
		0.8	CE: 52% Bio-HPR: 0.81 m^3^/m^3^/d C Bio-HR: 39% COD removal: 31%	[86]
	Acetate	0.6	Bio-HPR: 0.52 m^3^/m^3^/d	[87]
		0.8	Bio-HPR: 1.1 m^3^/m^3^/d	[87]
		1	Bio-HPR: 0.92 m^3^/m^3^/d	[89]
	Glucose	0.7	Bio-HPR: 1.63 m^3^/m^3^/d	[89]
9	Domestic wastewater	0.6	CE: 54% Bio-HPR: 0.50 m^3^/m^3^/d C Bio-HR: 25% COD removal: 12.4%	[89]
		0.8	CE: 57% Bio-HPR: 1.0 m^3^/m^3^/d C Bio-HR: 47% COD removal: 29.7%	[89]

C E: columbic efficiency, Bio-HPR: bio-hydrogen production rates, C Bio-HR: cathode bio-hydrogen recovery.

**Table 5:** Effect of temperature (°C) on performance of MEC.

Temperature (°C)	Substrate	Results	Ref.
20–25	Domestic wastewater	CE: 27.7% Bio-HPR: 0.3 L/d COD removal: 13.5%	[90]
25–30	Domestic wastewater	CE: 51.3% Bio-HPR: 0.5 L/d COD removal: 59.3%	[71]
30–35	Domestic wastewater	CE: 51.9% Bio-HPR: 0.3 L/d COD removal: 40.8%	[71]
	Industrial wastewater	Bio-HPR: 1.71 L/d	[88]
35–40	Domestic wastewater	CE: 44.7% Bio-HPR: 0.5 L/d COD removal: 12.6%	[90]
	Industrial wastewater	Bio-HPR: 2.51 L/d	[88]
40–45	Domestic wastewater	CE: 39.8% Bio-HPR: 0.3 L/d COD removal: 48.2%	[71]
	Industrial wastewater	Bio-HPR: 2.07 L/d	[90]
45–50	Domestic wastewater	CE: 42.4% Bio-HPR: 0.5 L/d COD removal: 41.0%	[71]
	Industrial wastewater	Bio-HPR: 1.62 L/d	[88]

C E: columbic efficiency, Bio-HPR: bio-hydrogen production rates.

**Table 6:** Effect of applied voltage (V) on performance of MEC.

Applied Voltage (V)	MEC Types	Substrate	Results	Ref.
0.2	Double chamber	Industrial wastewater	Bio-HPR: 1.7 m^3^/m^3^/d	[92]
		Fruit juice wastewater	Bio-HPR: 1609 m^3^/m^3^/d	[78]
	Single chamber	Food waste	Bio-HPR: 3.48 m^3^/m^3^/d	[93]
		Cellulose	Bio-HPR: 0.24 m^3^/m^3^/d	[91]
0.4	Double chamber	Acetate	CE: 78% Bio-HPR: 0.37 m^3^/m^3^/d C Bio-HR: 90% C D: 1 µA/cm^2^	[91]
0.5	Double chamber	Acetate	CE: 92% Bio-HPR: 0.02 m^3^/m^3^/d C Bio-HR: 57% C D: 1.9 µA/cm^2^	[92]
		Domestic wastewater	CE: 26% Bio-HPR: 0.01 m^3^/m^3^/d C Bio-HR: 42% C D: 2.5 µA/cm^2^	[81]
		Urban wastewater	CE: 40.3% Bio-HPR: 0.041 m^3^/m^3^/d C Bio-HR: 90%	[94]
	Single chamber	Glucose	CE: 127% Bio-HPR: 0.83 m^3^/m^3^/d C Bio-HR: 55%	[91]
		P-Glycerol	CE: 99% Bio-HPR: 0.8 m^3^/m^3^/d	[91]
		Industrial wastewater	Bio-HPR: 0.8 m^3^/m^3^/d	[72]
0.6	Double chamber	Acetate	CE: 64.00% Bio-HPR: 1.10 m^3^/m^3^/d	[94]
		Domestic wastewater	CE: 55% Bio-HPR: 0.015 m^3^/m^3^/d C Bio-HR: 70% C D:2.3 µA/cm^2^	[95]
	Single chamber	Proteins	CE: 75% Bio-HPR: 0.42 m^3^/m^3^/d C Bio-HR: 87% C D: 2.6 µA/cm^2^	[96]
		Acetate	CE: 73% Bio-HPR: 0.69 m^3^/m^3^/d C Bio-HR: 87% C D: 1.4 µA/cm^2^	[96]
		Molasses wastewater	CE: 93% Bio-HPR: 2.27 m^3^/m^3^/d	[97]
		Cassava starch processing wastewater	Bio-HPR: 0.46 m^3^/m^3^/d	[98]
0.7	Single chamber	Dairy wastewater	CE: 24% Bio-HPR: 0.2 m^3^/m^3^/d	[96]
0.8	Double chamber	Vegetable wastewater	Bio-HPR: 0.25 m^3^/m^3^/d C Bio-HR: 11.29% C D: 1.132 µA/cm^2^	[96]
		NaCl_2_	CE: 121.40% Bio-HPR: 1.300 m^3^/m^3^/d	[97]
	Single chamber	Milk	CE: 50% Bio-HPR: 0.086 m^3^/m^3^/d C Bio-HR: 91%	[96]
		Acetate	Bio-HPR: 3.12 m^3^/m^3^/d C Bio-HR: 96% C D: 1.7 µA/cm^2^	[96]
		Molasses wastewater	CE: 95% Bio-HPR: 1.82 m^3^/m^3^/d	[97]
		Glycerol, milk, starch	Bio-HPR: 0.94 m^3^/m^3^/d	[22]
		Industrial wastewater	Bio-HPR: 0.1 m^3^/m^3^/d	[91]
0.9	Double chamber	Glycerol	Bio-HPR: 3.9 m^3^/m^3^/d	[81]
		Aqueous phase of bio-oil	Bio-HPR: 4.3 m^3^/m^3^/d	[91]
	Single chamber	Sludge wastewater	CE: 78.89% Bio-HPR: 0.038 m^3^/m^3^/d C Bio-HR: 15.56–20.05%	[99]
		Glucose	CE: 105% Bio-HPR: 1.87 m^3^/m^3^/d	[97]
		P-Glycerol	CE: 104% Bio-HPR: 2.01 m^3^/m^3^/d	[97]
1.0	Double chamber	Acetate	Bio-HPR: 50 m^3^/m^3^/d C Bio-HR: 90% C D: 2.6 µA/cm^2^	[96]
		Industrial wastewater	CE: 16.82% Bio-HPR: 0.03 m^3^/m^3^/d C Bio-HR: 38.5%	[100]
	Single chamber	Acetate	Bio-HPR: 0.3 m^3^/m^3^/d	[101]
1.2	Double chamber	Glycerol	CE: 55% Bio-HPR: 0.46 m^3^/m^3^/d C Bio-HR: 85%	[94]
		Synthetic wastewater	CE: 21% Bio-HPR: 0.22 m^3^/m^3^/d	[97]
	Single chamber	Fermentation sludge	Bio-HPR: 0.16 m^3^/m^3^/d	[93]
1.5	Double chamber	Urban wastewater	CE: 28% Bio-HPR: 0.032 m^3^/m^3^/d	[97]

CE: columbic efficiency, C D: current density, Bio-HPR: bio-hydrogen production rates, C Bio-HR: cathode bio-hydrogen recovery.

**Table 7:** Effect of bacteria on performance of MEC.

MEC Types	Bacteria	Results	Ref.
Double chamber	*Hoeflea* sp.	Bio-HPR: 0.89 m^3^/m^3^/d	[104]
	*Shewanella oneidensis*	Bio-HPR: 61.8 m^3^/m^3^/d	[105]
	*Geobacter* sp.	Bio-HPR: 7.10 L/L/d C Bio-HR: ~100%	[106]
	*Geobacter sulfurreducens*	Bio-HPR: 0.133 m^3^/m^3^/d	[103]
Single chamber	*Geobacter Petrimonas*	Bio-HPR: 4.66 m^3^/m^3^/d	[107]
	*Geobacter sulfurreducens*	Bio-HPR: 4.18 m^3^/m^3^/d C Bio-HR: 89.3%	[107]
	*Dysgonomonas* sp.	Bio-HPR: 0.25–0.37 m^3^/m^3^/d	[105]
	*Rhodopseudomonas palustris*	Bio-HPR:0.44 mmol L/h	[102]
	*Pseudomonas aeroginosa*	Bio-HPR:14.3 mL/g of substrate	[108]

Bio-HPR: Bio-hydrogen production rates, C Bio-HR: Cathode Bio-hydrogen recovery.

## Data Availability

Not applicable.
